# Development of a droplet digital PCR for detection and quantitation of human parvovirus B19

**DOI:** 10.1128/spectrum.02522-25

**Published:** 2025-12-31

**Authors:** Xiaoyue Chu, Boya Zhao, Hailong Chen, Yuqi Jin, Linghao Zhang, Haichao Zheng, Na Feng, Jiacheng Chen, Zhe Zhao, Chaofeng Ma

**Affiliations:** 1 Shaanxi Blood Center 479107 , Xi'an, Shaanxi, China; 2 Shaanxi University of Chinese Medicine 107652, Xianyang, Shaanxi, China; 3 Xi’an Center for Disease Control and Prevention, Xi'an, Shaanxi, China; 4 School of Public Health, Health Science Center, Xi'an Jiaotong University 599469, Xi'an, China; Ascension St John Hospital, Detroit, Michigan, USA

**Keywords:** human parvovirus B19, droplet digital PCR, NS1 region, nucleic acid testing

## Abstract

The objective of this study is to establish a droplet digital PCR (ddPCR) method for the detection of human parvovirus B19 (B19V) and provide accurate and reliable technical support for molecular biological diagnosis and epidemiological investigation of the virus. Specific primers and a TaqMan probe targeting the NS1 region of the B19V genome were designed, and a reaction system based on ddPCR was constructed and optimized. The methodology was validated through sensitivity, specificity, and repeatability tests. Subsequently, the method was applied to eight B19V-positive samples to evaluate its practical applicability. Methodological validation experiments demonstrated excellent sensitivity with a good linear relationship (*R*² = 0.9974) and a minimum detection limit of 1.013 × 10⁻¹ copies/μL. No cross-reactivity was observed with 11 respiratory pathogens, 5 pathogens presenting similar clinical manifestations, and 4 blood-borne pathogens. The inter- and intra-assay coefficients of variation for high-, medium-, and low-concentration samples were all ≤10%. Furthermore, this method was successfully applied to clinical samples, with stable detection of B19V in five high-concentration throat swab samples and three low-concentration blood samples. The ddPCR method established in this study exhibits high sensitivity, specificity, and repeatability, offering a robust tool for molecular diagnosis and epidemiological monitoring of B19V infection.

**IMPORTANCE:**

Early human parvovirus B19 (B19V) infections were sporadic or occurred in small clusters, attracting little attention. Since late 2023, nine European Union/European Economic Area (EU/EEA) countries have reported a significant increase in B19 infections. Moreover, the virus has robust physicochemical tolerance and the potential to resist pathogen removal processes such as filtration, inactivation, and pasteurization, which have raised the close attention and vigilance of international organizations, governments, and the public. Despite existing qPCR and antigen/antibody tests, the growing number of infections in multiple countries highlights the need for a more accurate and efficient detection system. Based on this, our team carried out related research and built a system configuration based on the third-generation droplet digital polymerase chain reaction platform, with a view to updating the B19V detection method.

## INTRODUCTION

As the sole virus within the genus Erythrovirus of the subfamily Parvovirinae, known to cause disease in humans, human parvovirus B19 (B19V) has a diameter of approximately 25 nm and comprises a small, non-enveloped, single-stranded linear DNA genome of about 5,596 nucleotides (1). The virus is transmitted worldwide via the respiratory tract, blood/blood products, and the placenta (2). Clinical manifestations of B19V infection are closely related to the host’s age, hematologic status, and immune status. Healthy children and adults are typically asymptomatic or exhibit only mild, non-specific symptoms. Typical presentations include erythema infectiosum (also known as “fifth disease”), symmetric rashes, and arthralgia. In contrast, infection in immunocompromised individuals (3), patients with hematologic disorders (4), organ transplant recipients (5), pregnant women, and fetuses may lead to severe complications, such as acute or chronic immune thrombocytopenic purpura, pure red cell aplasia, multiple autoimmune diseases, and adverse pregnancy outcomes, which can even result in death (6, 7).

At present, polymerase chain reaction (PCR) is widely used for nucleic acid testing and has evolved through three generations: from traditional PCR for qualitative analysis, to semiquantitative real-time fluorescence quantitative PCR (qPCR), and finally to absolute quantitative digital PCR (dPCR) (8). Among these, qPCR is currently the mainstream detection method, and a universal nucleic acid detection system for the three genotypes of B19V based on this method has been established in relevant literature (9). Numerous such nucleic acid detection kits are available on the market (e.g., Roche cobas TaqScreen DPX Test). However, robust dPCR-based assays for B19V are limited, and none have been rigorously optimized and independently validated (10, 11). To address this gap, we developed and systematically evaluated a droplet digital PCR (ddPCR) protocol. The findings are reported as follows.

## MATERIALS AND METHODS

### Sample source

Novel coronavirus (SARS-CoV-2), *Streptococcus pneumoniae*, common coronavirus (HCoV), parainfluenza virus (HPIV), respiratory adenovirus (HAdV), influenza A virus (FluA), respiratory syncytial virus (RSV), rhinovirus (HRV), human bocavirus (HBoV), *Mycoplasma pneumoniae*, enterovirus (EV), and five high-titer B19V isolates were recovered from throat swab samples. Human immunodeficiency virus (HIV), hepatitis B virus (HBV), hepatitis C virus (HCV), *Treponema pallidum*, and three low-titer B19V isolates were obtained from blood samples. Epstein–Barr virus (EBV), human herpesvirus 6 (HHV-6), varicella-zoster virus (VZV), rubella virus, and measles virus were used as commercially obtained quality-control materials.

### Reagents and equipment

In this study, the GeneRotex 96 automatic nucleic acid extractor and the corresponding kit from Tianlong Technologies (Xi'an) Co., Ltd., were used to extract RNA/DNA from the samples. The 4× RT dPCR Mix, Probe dPCR HiTaqMix (with UNG), microfluidic chip, sealing oil, and the automatic droplet digital PCR instrument required for the ddPCR reaction were purchased from Xinyi Biological Manufacturing (Beijing) Co., Ltd.

### Establishment, optimization, and verification of methods

#### Design and synthesis of primers and probes

Based on the complete B19V genome sequence in NCBI (GenBank NC_000883.2), the highly conserved NS1 region was identified by BLAST alignment and used to design primers and probe with Primer Premier 5.0. The 121 bp amplicon is flanked by forward primer B19-F (5′-CGGGACCAGTTCAGGAGAAT-3′, nt 2,253–2,272) and reverse primer B19-R (5′-CCCAACTAACAGTTCACGAAACT-3′, nt 2,351–2,373). The TaqMan probe B19-P (5′−6-FAM-TCGGAAGCCCAGTTTCCTCCGA-BHQ1-3′, nt 2,279–2,300) was positioned between the primers. All oligonucleotides were synthesized by Sangon Biotech (Shanghai) Co., Ltd., with a working concentration of 10 μM each.

#### Design and synthesis of positive plasmid standards

Based on the results of multiple sequence alignment, a recombinant plasmid containing the conserved region NS1-VP1 (2,078–2,959) of the B19V was constructed and synthesized by GenScript (Beijing) Co., Ltd. The synthesized plasmid was diluted to 40 μL with nuclease-free water to a concentration of 4 μg as the original standard solution for subsequent experiments. The concentration of the standard solution was calculated using the formula (dry weight × Avogadro’s number) / (average molecular weight of 1 bp × total plasmid length × diluted vol), resulting in a concentration of 1.013 × 10^10^ copies/μL (100 ng/μL).

#### Optimization of the reaction system

The concentrations of primers and probes are key factors affecting the ddPCR detection results. The standard solution was diluted to 1.013 × 10^4^ copies/μL as the DNA template. A matrix method was employed to optimize the addition amounts of B19-F/R (final concentrations of 400, 600, and 800 nM) and B19-P (final concentrations of 100, 200, 300, and 400 nM). In principle, the final concentration of the probe should be slightly lower than that of the primers. Therefore, eight combinations that met this criterion were selected for experimentation to obtain the optimal reaction system.

#### Sensitivity and limit of detection assays

A dilution series of the plasmid standard was prepared to assess the linearity of the assay: 202,600, 151,950, 101,300, 50,650, 25,325, 12,662.5, 2,532.5, 1,266.25, 253.25, 126.625, 25.325, 12.6625 copies/5 μL. A linear relationship between theoretical copy numbers (*x*) and measured values (*y*) was established using the equation *y* = mx + *b* to evaluate the sensitivity of the method. Subsequently, the lowest concentration was further diluted serially (10- and 5-fold) and tested until the *N*-th group showed a positive result while the (*N* + 1)-th group showed a negative result. The *N*-th and (*N* + 1)-th groups were tested in triplicate to confirm reproducibility and to evaluate the limit of detection of the method.

#### Specificity assay

A plasmid standard (1.013 × 10⁴ copies/μL) and ddH_2_O served as positive and negative controls, respectively. To assess specificity, the optimized system was applied to nine non-RT DNA pathogens (HAdV, HBoV, HBV, *M. pneumoniae*, *S. pneumoniae*, *T. pallidum*, EBV, HHV-6, and VZV). Eleven RNA pathogens (SARS-CoV-2, HCoV, HPIV, FluA, RSV, HRV, EV, HIV, HCV, rubella virus, and measles virus) were tested with 4× RT dPCR Mix (7.5 μL) for reverse transcription and amplification.

#### Repeatability assay

The plasmid standard solution was serially diluted to three concentrations: 1.013 × 10^4^ copies/μL, 1.013 × 10^3^ copies/μL, and 1.013×10^2^ copies/μL, which were used as high-, medium-, and low-concentration templates, respectively. These templates were aliquoted and stored at −20°C for later use. The repeatability assay included intra-assay repeatability and inter-assay repeatability. The intra-assay repeatability was assessed by testing each concentration sample three times using the optimized system at the same time point. The inter-assay repeatability was evaluated by conducting three independent experiments under the same conditions at different time points. The mean ($\bar{x}$), standard deviation, and coefficient of variation (CV) were calculated from the detection results to evaluate the repeatability of the method.

#### Clinical sample detection

The ddPCR assay developed here was applied to eight B19V-positive specimens (five respiratory throat swab and three blood samples) collected from patients and voluntary blood donors in Shaanxi Province between August 2024 and May 2025. Nucleic acid extraction was carried out strictly according to the kit manufacturer’s instructions.

### Result judgment and data analysis

The reaction was conducted using an automated ddPCR instrument, along with its accompanying Drop Maker sample preparation system and Chip Reader biochip analysis system, for the generation, preparation, reading, and analysis of nanoliter-volume “water-in-oil” droplets. The amplification results were judged based on the presence and quantity of droplets. An Excel 2019 database was established, and statistical analysis was performed using SPSS 27.0 software. The mean and standard deviation were represented as $\bar{x}$ ± *s*. Linear fitting and graphing were carried out using Origin 2019b.

## RESULTS

### Optimization results of the reaction system

**Fig 1:**
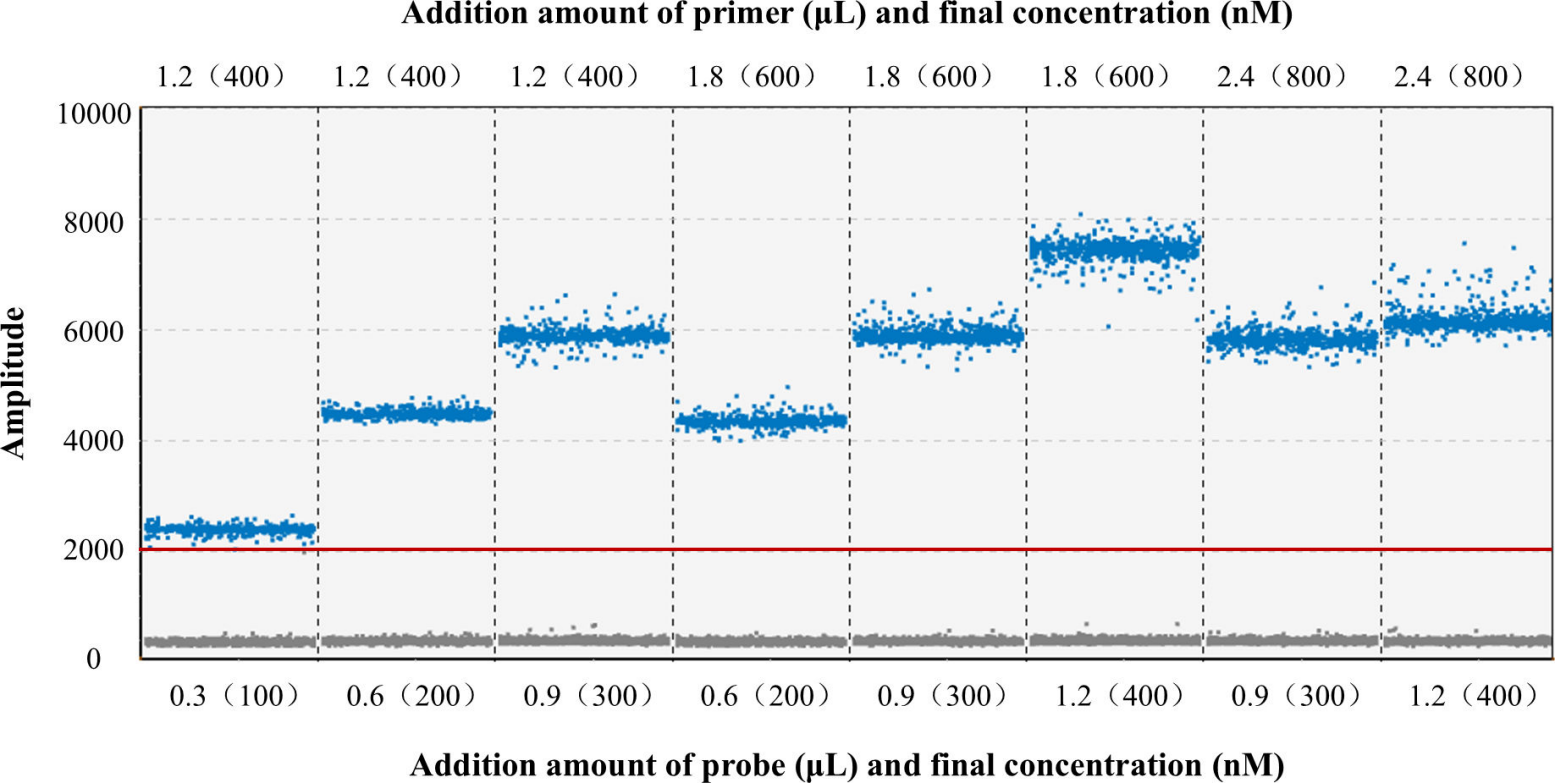
Optimization of the reaction system: FAM fluorescence plot. The figure compares the detection results obtained with different combinations of primer (upper values) and probe (lower values) volumes (μL) and their final concentrations (nM). Taking both amplification efficiency and cost into account, the combination (1.2 μL primer/400 nM, 0.9 μL probe/300 nM) showed the clearest separation between positive and negative droplets and the great fluorescence difference and was therefore selected as the optimal reaction condition.

The ddPCR detection results are shown in Fig. 1. The optimal detection effect was achieved with a primer volume of 1.8 μL (final concentration of 600 nM) and a probe volume of 1.2 μL (final concentration of 400 nM). However, considering the economic cost and the fluorescence intensity, the experimental group with lower concentrations and better fluorescence intensity is more practical. When the primer volume was 1.8 μL (final concentration of 400 nM) and the probe volume was 0.9 μL (final concentration of 300 nM), the gap between positive and negative droplets was significant, with a high fluorescence intensity difference of 5,600. Therefore, this study selected this configuration to construct the method.

The final amplification system was determined as follows: Probe dPCR HiTaqMix 7.5 μL (6 μL in tube A and 1.5 μL in tube B), 1.2 μL of each 10 μM upstream and downstream primer (both with a final concentration of 400 nM), 0.9 μL of 10 μM probe (final concentration of 300 nM), 5 μL of template, and nuclease-free water to a final volume of 30 μL. The amplification program was as follows: rapid amplification with a 30-second run at 95°C, 10 seconds of denaturation at 94°C, and 30 seconds of annealing and extension at 58°C for 40 cycles.

### Sensitivity assay and limit of detection results

**Fig 2:**
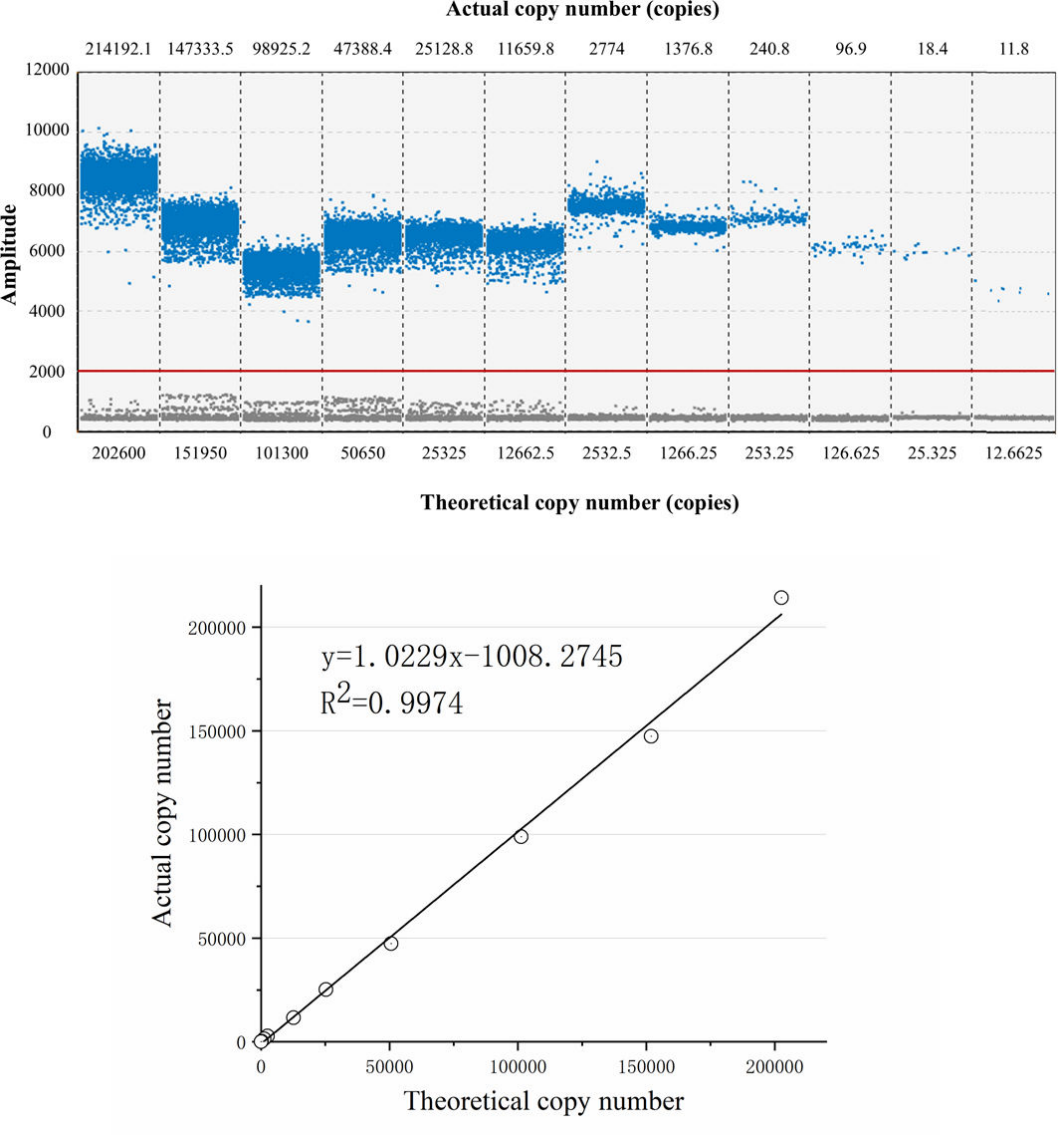
Sensitivity assay: FAM fluorescence plot and linear fit. B19V plasmid standards were serially diluted from 12,662.5 to 202,600 copies/μL and analyzed by ddPCR, and the results are shown in the upper panel. A linearity plot (lower panel) was generated with theoretical copy numbers on the *x*-axis and measured copy numbers on the *y*-axis. The fitted line (dashed) yielded the equation *y* = 1.0229*x* – 1,008.3 (*R*² = 0.9974).

As shown in Fig. 2, the copy numbers of the 12 dilution groups decreased progressively with the dilution of the B19 plasmid standard, with values of 214,192.1, 147,333.5, 98,925.2, 47,388.4, 25,128.8, 11,659.8, 2,774, 1,376.8, 240.8, 96.9, 18.4, and 11.8 copies, respectively. A calibration curve was plotted with theoretical copy numbers on the *x*-axis and actual copy numbers on the *y*-axis, yielding a linear equation: *y* = 1.0229*x* − 1,008.2745, with a correlation coefficient (*R*^2^) of 0.9974, indicating good sensitivity of the method.

The limit of detection was determined by testing samples at progressively lower concentrations. When the concentration was 1.013 × 10^−1^ copies/μL, positive droplets were still detected with results of 5.7, 2.8, and 1.4 copies. In contrast, at the next lower concentration of 5.065 × 10^−2^ copies/μL, no positive droplets were detected, with copy results of 0. Therefore, the limit of detection of the method was established at 1.013 × 10^−1^ copies/μL.

### Specificity assay results

Specific amplification occurred exclusively in the B19V-positive control; none of the other 20 pathogen panels (11 respiratory, 5 phenotypically similar, and 4 blood-borne pathogens) or the negative control produced positive droplets, confirming the high specificity of the assay.

### Repeatability assay results

As shown in Table 1, the CV increased with the dilution of the plasmid concentration. For each plasmid concentration, the inter-assay CV was greater than the intra-assay CV. The intra-assay CVs for the high-, medium-, and low-concentration groups were 2.7%, 4.3%, and 8.4%, respectively, while the inter-assay CVs were 3.2%, 9.8%, and 10.0%, respectively. All CVs were less than or equal to 10%, indicating good repeatability of the detection method.

**TABLE 1:** Intra-assay and inter-assay repeatability test results^*a*^

Plasmid concentration (copies/μL)	Intra-assay repeatability			Inter-assay repeatability		
	Copy number (copies)	Mean ± standard deviation ($\bar{x}$ ± *s*)	CV (%)	Copy number (copies)	Mean ± standard deviation ($\bar{x}$ ± *s*)	CV (%)
1.013 × 10^4^	7,782.2	7,984.5 ± 218.5	2.7	7,955.0	7,945.6 ± 253.3	3.2
	8,216.3			8,194.1		
	7,955.0			7,687.8		
1.013 × 10^3^	754.0	790.0 ± 33.8	4.3	794.9	729.2 ± 71.3	9.8
	794.9			739.4		
	821.1			653.4		
1.013 × 10^2^	22.3	23.6 ± 1.4	8.4	25.1	28.3 ± 2.8	10.0
	23.4			29.3		
	25.1			30.5		

^*a*^ CV, coefficient of variation.

### Clinical sample detection results

**Fig 3:**
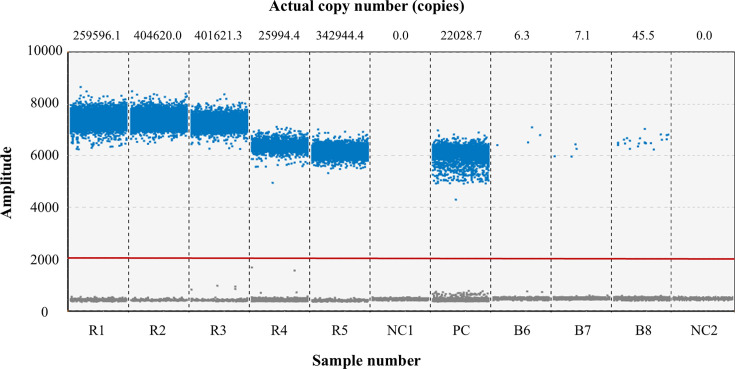
Detection of positive samples: FAM fluorescence plot. Copy numbers are plotted for each specimen. The PC group serves as the positive control (plasmid standard); the NC1 and NC2 groups are negative controls derived from respiratory and blood sources, respectively; samples R1–R5 originate from throat swabs; samples B6–B8 originate from blood.

Using the ddPCR method established in this study, we tested eight B19V-positive samples. The results, as shown in Fig. 3, indicate that five high-concentration throat swab samples (ranging from 259,596.1 to 404,620.0 copies/5 μL) and three low-concentration blood samples (ranging from 6.3 to 45.5 copies/5 μL) were all reproducibly detected. These findings demonstrate that the method is applicable to actual clinical samples.

## DISCUSSION

Epidemiological studies and clinical diagnosis of B19V rely on serological and molecular approaches, involving *in vitro* detection of B19V antigen, IgM/IgG antibodies, and nucleic acids derived from human samples. Antigen detection exhibits low sensitivity, often resulting in false-negative results (12). The IgM antibody, serving as a marker for acute infection and early diagnosis, typically emerges in the late stage of viremia (around 7–10 days). In contrast, IgG antibodies, indicative of long-term infection, begin to appear approximately 15 days post-infection and may persist at high titers for several months or even lifelong (13). However, studies have demonstrated that immunosuppression, cross-reactivity, and transient viremia can limit the reliability of these methods, necessitating the use of molecular biological detection results for corroboration (14–16).

Presently, ddPCR as the latest generation of nucleic acid detection methods works by randomly distributing limited-dilution target sequences into tens of thousands of micro-reactions. After single-molecule template PCR amplification, the endpoint fluorescence signals of each micro-reaction are detected and interpreted. The target concentration is then absolutely quantified using Poisson distribution principles (17). Compared to first- and second-generation PCR techniques, digital PCR offers significant advantages in infectious virus detection. It does not rely on standards or standard curves, enabling absolute quantification and facilitating the comparison and sharing of results across different laboratories. Its amplification efficiency is less affected by primers or templates, resulting in higher accuracy. Moreover, digital PCR is more sensitive, particularly when detecting extremely low-abundance targets, rare mutations in complex backgrounds, or minor changes in nucleic acid copy numbers (18–23). Consequently, ddPCR has become a key enabler in life-science research and molecular diagnostics.

Based on available literature reports, only Zulli et al. (10) and Das et al. (11) have attempted to detect B19V in cardiac tissue or sewage-derived samples by ddPCR; however, neither team performed independent, systematic re-validation or performance optimization of their assays. Owing to its small size and high physicochemical stability, parvovirus B19 is transmitted mainly via the respiratory route or through contaminated blood/blood products and can infect immunocompromised individuals (24). We therefore optimized and comprehensively evaluated a ddPCR method for the detection and absolute quantification of human parvovirus B19 and verified its suitability for both respiratory throat swab and blood specimens. Method validation revealed a linear range of 202,600–12.66 copies/5 µL (*R*² = 0.9974) and a limit of detection of 1.013 × 10⁻¹ copies/µL, which is lower than that of the B19V-qPCR system (10 copies/µL) reported by Jia et al. (25). No cross-reactivity was observed with 20 other pathogens; intra- and inter-assay CVs for high, medium, and low concentrations were ≤10%. The assay was further applied to eight B19V-positive clinical samples (five high-titer throat swabs and three low-titer blood samples), all of which were correctly detected, demonstrating its excellent sensitivity, specificity, repeatability, and clinical applicability.

In summary, the ddPCR detection method for B19V constructed in this study aims to provide technical support and assurance for early clinical detection of the virus, molecular biological diagnosis of minor target concentration changes, monitoring of virus safety in blood transfusions and blood products, and epidemiological investigations.

## Data Availability

No novel sequence data were generated in this study. All genomic sequences analyzed are publicly available through the NCBI databases under the accession numbers listed in the manuscript.

## SUPPLEMENTAL MATERIAL

Figure S1 spectrum.02522-25-s0001.docx No cross-reactivity was observed with any of the 20 pathogens tested (including respiratory, blood-borne, and clinically similar agents), demonstrating the high specificity of the assay.

Figure S2 spectrum.02522-25-s0002.docx We re-tested the eight B19V-positive samples included in this study by qPCR. As shown in the figure, the five respiratory throat swab samples exhibited low Ct values with amplification curves close to that of the positive control, whereas the three blood-derived samples with low viral loads gave high Ct values. This trend is fully consistent with the quantitative results obtained by ddPCR.

ASM does not own the copyrights to Supplemental Material that may be linked to, or accessed through, an article. The authors have granted ASM a non-exclusive, world-wide license to publish the Supplemental Material files. Please contact the corresponding author directly for reuse.
