# Supplementary material for: Development of a droplet digital PCR for detection and quantitation of human parvovirus B19
Source: Microbiol Spectr. 2025 Dec 31;14(2):e02522-25. doi: 10.1128/spectrum.02522-25 (PMC12889099; doi:10.1128/spectrum.02522-25)
Supplement: Figure S1 — No cross-reactivity was observed with any of the 20 pathogens tested (including respiratory, blood-borne, and clinically similar agents), demonstrating the high specificity of the assay. [file spectrum.02522-25-s0001.docx]

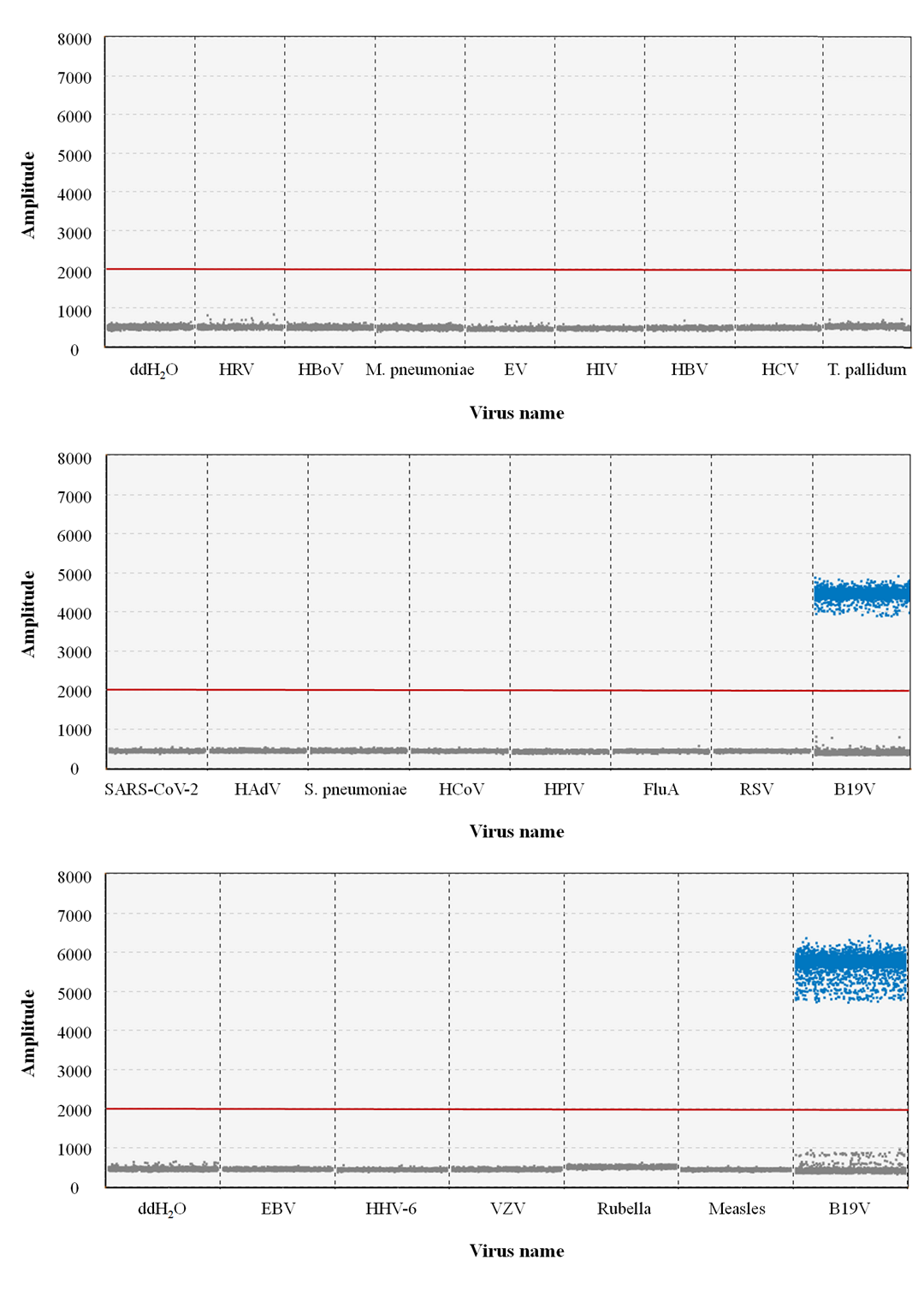


**Supplementary material 1: Specificity Assay: FAM Fluorescence Plot.** As shown in the figure, no cross-reactivity was observed with any of the 20 pathogens tested (including respiratory, blood-borne, and clinically similar agents), demonstrating the high specificity of the assay.
