# Supplementary material for: Development of a droplet digital PCR for detection and quantitation of human parvovirus B19
Source: Microbiol Spectr. 2025 Dec 31;14(2):e02522-25. doi: 10.1128/spectrum.02522-25 (PMC12889099; doi:10.1128/spectrum.02522-25)
Supplement: Figure S2 — We re-tested the eight B19V-positive samples included in this study by qPCR. As shown in the figure, the five respiratory throat swab samples exhibited low Ct values with amplification curves close to that of the positive control, whereas the three blood-derived samples with low viral loads gave high Ct values. This trend is fully consistent with the quantitative results obtained by ddPCR. [file spectrum.02522-25-s0002.docx]

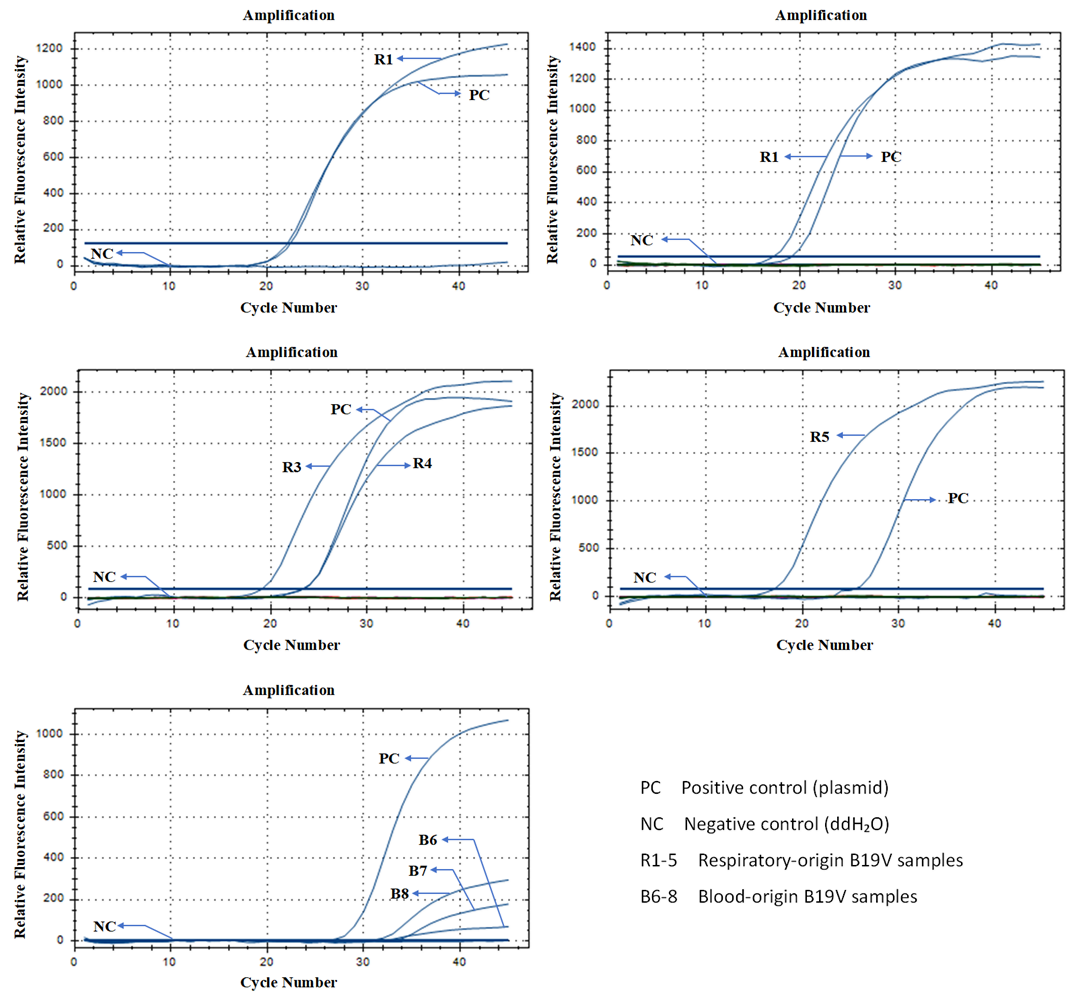


**Supplementary material 2：Detection of positive samples by qPCR method.** We re-tested the eight B19V-positive samples included in this study by qPCR. As shown in the figure, the five respiratory throat-swab samples exhibited low Ct values with amplification curves close to that of the positive control, whereas the three blood-derived samples with low viral loads gave high Ct values. This trend is fully consistent with the quantitative results obtained by ddPCR.
